# Examination of genetic variants involved in generation and biodisposition of kinins in patients with angioedema

**DOI:** 10.1186/s13223-014-0060-y

**Published:** 2014-12-12

**Authors:** Jonathan Levy, Georges-Etienne Rivard, Eric Wagner, Don Beezhold, Noam Berlin, Li Fan, Zhao Zhang, Gordon L Sussman

**Affiliations:** Division of Dermatology and Cutaneous Sciences, University of Alberta, 2-166 Clinical Sciences Building, 11350 – 83 Avenue, Edmonton, Alberta T6G 2G3 Canada; CHU Sainte-Justine, Université de Montréal, Montreal, QC Canada; CHU de Québec and UniversitéLaval, Quebec City, QC Canada; National Institute for Occupational Safety and Health, Morgantown, WV Canada; University of Toronto, Toronto, ON Canada

**Keywords:** Angioedema, Plasminogen-activator inhibitor-1, Aminopeptidase P, Angiotensin-converting enzyme, Factor XII

## Abstract

**Background:**

Angioedema (AE) is idiopathic in the majority of cases. We studied patients with AE for genetic variants of proteins involved with bradykinin generation and biodisposition.

**Methods:**

One hundred sixty one patients with AE were recruited at a university hospital clinic. Patients were categorized according to the proposed pathogenesis of AE: low C1 inhibitor (C1-INH) and C4 levels, autoimmune disease, cancer, angiotensin-converting enzyme (ACE) inhibitor-induced, nonsteroidal antiinflammatory drug (NSAID)-induced, or idiopathic. In addition, each patient had a blood sample analyzed for a complement profile and enzymes (C1-INH and C4). Fifty-two of the patients were tested for genetic variants in factor XII, plasminogen-activator inhibitor-1 (PAI-1), ACE, and aminopeptidase P (APP).

**Results:**

The cause of angioedema was identified in 59/161 (37%) of the cases: 3 (2%) patients had a low plasma C1-INH and C4; 20 (12%) were ACE inhibitor-induced; 12 (7%) were associated with autoimmune disorders; 7 (4%) were associated with malignancy; and 17 (11%) were associated with NSAIDs. In the remaining 102 (63%) patients the cause of angioedema was idiopathic. Of 52 patients with genetic analysis, 13 (25%) had a genetic variant in APP, 10 (19%) in ACE, 13 (25%) in PAI-1, and 0 in Factor XII.

**Conclusions:**

In addition to related diseases and medications causing AE, certain genetic variants encoding proteins involved in bradykinin generation and/or catabolism pathways may be involved in the pathogenesis of AE.

## Background

Angioedema (AE) is a self-limited swelling in the dermis, subcutaneous tissue, mucosa, and submucosa that can last for hours to days [1]. It is a potentially life-threatening disease in cases of laryngeal AE. The pathophysiology differs from urticaria, which involves the epidermis and dermis. Several variants of angioedema exist making diagnosis and initiating appropriate treatment difficult.

AE can be broadly categorized as those presenting with and without associated urticaria. AE with urticaria is occasionally allergic and due to food, medication, latex, insect venom, or radiocontrast media [2], but many cases are idiopathic [3]. AE without urticaria can be non-histamine mediated due to C1 inhibitor (C1-INH) deficiency (hereditary or acquired), or related to angiotensin converting enzyme (ACE) inhibitor use, autoimmune diseases, malignancies, or nonsteroidal anti-inflammatory drug (NSAID) use, but in many cases is idiopathic.

Hereditary angioedema (HAE) is caused by mutations in *Serping1*, which encodes C1-INH, a serine protease inhibitor that regulates activation of the classical and lectin (and possibly the alternative) complement pathways and the contact activation pathway of the coagulation system [4]. Pattern of inheritance is autosomal dominant in the vast majority of affected patients who generally have partial C1-INH deficiency [5,6] and cannot efficiently control the contact activation system. Type I HAE is due to low circulating levels of functional C1-INH, whereas type II is due to normal to high levels of non-functional C1-INH. Recently, HAE with normal C1-INH (type III) has been described as an estrogen-related hereditary form with normal functional levels of C1-INH and affecting predominantly women who sometimes have a gain of function variant of the gene coding for coagulation factor XII [7]. An acquired form of C1-INH deficiency also leads to AE and is seen in patients with autoimmune disease or certain malignancies [8]. Diagnosis of acquired C1-INH deficiency requires a negative family history and its onset is usually after the 4^th^ decade of life, in contrast to hereditary C1-INH deficiency. It is associated occasionally with antibodies that react in vitro to C1-INH. AE due to ACE inhibitor or NSAID use may present without urticarial involvement or in concurrence with chronic spontaneous urticaria, which is defined as urticaria occurring for at least six weeks due to an endogenous cause and not external physical stimuli [9]. In many patients, AE occurs in the absence of any known cause [10].

**Figure 1:**
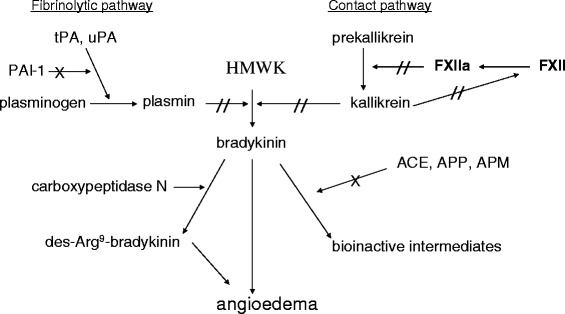
**The fibrinolytic and contact pathways demonstrating the generation and catabolism of bradykinin, which clinically may present as angioedema.** HMWK, high molecular weight kininogen; tPA, tissue plasminogen activator; uPA, urokinase-like plasminogen activator; PAI-1, plasminogen activator inhibitor-1; FXII, factor XII; ACE, angiotensin converting enzyme; APP, aminopeptidase P; APM, aminopeptidase M. The effect of C1inh was not included. //, blocked by C1-INH, C1 inhibitor; X, negative regulation.

Non-histaminergic angioedema is most likely caused by the generation of bradykinin, a potent vasoactive peptide [11,12]. Bradykinin is generated mainly through activation of the contact system (Figure 1). Upon activation, factor XII cleaves prekallikrein into kallikrein, which in turn cleaves high molecular weight kininogen to free the potent bradykinin peptide. Another pathway through which bradykinin can be generated is via the fibrinolysis pathway, although at a lesser extent. Indeed, plasmin, generated from plasminogen by the action of the plasminogen activators, tissue plasminogen activator and urokinase-like plasminogen activator, can cleave high molecular weight kininogen into bradykinin. Activity of tissue plasminogen activator and urokinase-like plasminogen activator is inhibited by plasminogen-activator inhibitor-1 (PAI-1) [13]. Bradykinin is short-lived and rapidly transformed by carboxypeptidase N into a bioactive intermediate des-Arginine-9 bradykinin and/or bioinactive intermediates by ACE and aminopeptidases P (APP) [14] and M.

We hypothesize that defects in factors involved in bradykinin generation or its catabolism may be associated with AE attacks. This has been demonstrated in patients who present with AE with neither C1-INH deficiency nor any other known cause [10]. Some patients exhibit gain-of-function mutations in factor XII that are suggested to lead to increased activity, therefore increasing bradykinin generation upon contact system activation [15]. Also, polymorphisms affecting genes encoding ACE and APP have been associated with increased levels of bradykinin and/or des-Arg-9 bradykinin presumably related to reduced biodegradation [16]. Occurrence of these polymorphisms in addition to mutations in factor XII has been shown in patients with estrogen-related AE [17]. Polymorphisms in *XPNPEP2,* which encodes APP, have been associated with ACE inhibitor-associated AE [18,19]. Further, polymorphisms in PAI-1 are known in humans [20] that theoretically could be associated with AE in some patients by allowing increased generation of plasmin. The 5G variant is associated with less inhibition of plasminogen activators and, consequently, increased conversion of plasminogen to plasmin [21] and potentially more generation of bradykinin.

We studied patients with AE by genetic analysis for variants in genes encoding proteins involved in bradykinin generation (factor XII, PAI-1) and enzymes involved in bradykinin catabolism (ACE, APP). A further objective was to classify patients according to etiology: decreased levels of C1-INH and C4, ACE inhibitor-induced, autoimmune disease, malignancy, NSAID-induced, or idiopathic.

## Methods

One hundred sixty-one patients presenting with AE at a university hospital clinic were recruited. Diagnosis of AE was made by an expert physician (GLS) based on clinical history and direct physical examination. All patients provided informed written consent and ethics approval was received through a research ethics board (Canadian SHIELD, Burlington, ON, Canada). We obtained a detailed clinical history including the location of AE (central, peripheral, laryngeal, and/or abdominal), family history of AE and other autoimmune disorders, malignancies, autoimmune diseases, use of ACE inhibitor or NSAID, and associated urticaria. Routine skin prick tests to common airborne and environmental allergens were done (ALK-Abelló, Port Washington, NY). Each patient had a blood sample analyzed for antigenic and functional levels of C1-INH and antigenic levels of C4. C1-INH levels were measured either by immunonephelometry (Beckman Coulter, Brea, CA) or radial immunodiffusion (The Binding Site, San Diego, CA) and C4 levels were assessed using immunonephelometry (Beckman Coulter). C1-INH function was measured using a standard chromogenic assay (Berichrom C1-INH, Siemens, Erlangen, Germany). Patients were then categorized according to the proposed pathogenesis of AE: low C1-INH and C4 levels, ACE inhibitor-induced, autoimmune disease, malignancy, NSAID-induced, or idiopathic. Blood specimens suitable for DNA extraction were available from 52 of the 161 patients. They were tested for specific genetic variants in genes encoding proteins or enzymes involved in direct or indirect regulation of bradykinin generation and catabolism, specifically factor XII, PAI-1, ACE, and APP. DNA samples were analysed by standard molecular techniques looking for the following genetic variants: factor XII (mutation c.1032C > A or c.1032C > G), PAI-1 (4G/5G polymorphism), ACE (insertion/deletion I/D polymorphism, Single Nucleotide Polymorphism Database (dbSNP): rs1799752), APP (deletion g.2953-3127del, single nucleotide polymorphism (SNP) c.-2399C > A, dbSNP: rs3788853).

Whole blood was collected in EDTA and stored at −80°C until used. Genomic DNA was extracted with the Qiagen DNA extraction kit (Qiagen, California, USA). Each sample was tested for 6 target genetic variants in 4 different genes (XPNPEP2, ACE, F12, Serpine 1).

### Polymorphism and deletion in Aminopeptidase P

For Aminopeptidase P (APP), the gene XPNPEP2 is located in chromosome Xq26.1. A polymorphism (c.-2399C > A) is in the promoter region, while a 175-bp deletion is in exon 2 (g.2953-3127del) [22].

The primers for c.-2399C > A were: forward APP-SNP-2 F 5′-TCC CCG TTT AGT TTG TTT GC-3′, reverse APP-SNP-1R 5′-GGG CTA ATG TTG GTG ATG CT-3′. 100 ng genomic DNA was subjected to PCR with 200 μM of dNTP, 1.5 mM of MgCl2, 1 μM of primers, 1.25U of Taq DNA polymerase Gold (ABI, Burlington, ON, Canada), 1X buffer in a final volume of 25 μl in ABI 9700 thermal cycler. The DNA was denatured at 95°C for 8 min followed with 35 cycles of 94°C for 50 sec, 56°C for 50 sec, and 72°C for 1 min. The extension is at 72°C for 10 min. Amplicons of PCR were sequenced to detect this polymorphism.

The primers for g.2953-3127del were: forward APPdel-3 F1 (5′-TTT CTC CCG GCT TCT AGC TT-3′), and reversed primer APPdel-3R0 (5′-CTC AGC CAA AGG CCA GTT AG-3′). 150 ng genomic DNA is subject to PCR with 400 μM of dNTP, 0.8 μM of primers, 1 M of betaine and 1.6U of long bp Taq DNA polymerase ELT (Roche, Indianapolis, IN, USA), 1X buffer #2 in a final volume of 30 μl. The DNA was first denatured at 95°C for 7 min, cooled on ice, then with brief centrifugation, followed by 35 cycles of 94°C for 3 min, 94°C for 50 sec, 63°C for 50 sec, 72°C for 1 min, and extension at 72°C for 10 min.

### ACE polymorphism

The ACE gene encodes the angiotensin-converting enzyme (ACE). An insertion/deletion (I/D) polymorphism is located in intron 16 of the gene. This polymorphism is characterized by the presence (called I allele) or absence (called D allele) of a 287 bp Alu repeat sequence. In Caucasians, the I allele is associated with lower, and the D allele with higher circulating ACE activity, respectively. The heterozygotes for insertion/deletion (I/D) present about one-half of the variance of the activity [23].

Forward primer ACE-I/D-F was 5′-CTG GAG AGC CAC TCC CAT CCT TTCT-3′, reverse primer ACE-I/D-R is 5′- GAC GTG GCC ATC ACA TTC GTC AGA -3′.

150 ng genomic DNA was subject to PCR with 400 μM of dNTP, 0.8 μM of primers ACE-I/D-F and ACE-I/D-R, 1 M of betaine and 1.6U of long bp Taq DNA polymerase ELT, 1X buffer #2 (Roche, Indianapolis, IN, USA) in a final volume of 30 μl. The DNA was denatured at 95°C for 7 min, cooled on ice before brief centrifugation, followed by 35 cycles of 94°C for 3 min, 94°C for 50 sec, 63°C for 50 sec, 72°C for 1 min, and extension at 72°C for 10 min.

### Factor XII mutation

The F12 gene encodes coagulation factor XII (Hageman factor). Mutations c.1032C > A and c.1032C > G are located in exon 9 of the F12 gene. Forward primer FXII-ex9-F2 was 5′-CTG GGA GTA CTG CGA CCT G-3′, reverse primer FXII-ex9-R1 was 5′- AAG GCT GTG GAG GAG CAG -3′. One hundred fifty ng no of genomic DNA DNA was subject to PCR with 200 μM of dNTP, 1 μM of primers, 1.5 mM of MgCl2, 1.25 U of Taq DNA polymerase (ABI), 1X buffer, in a final volume of 30 μl. The PCR reaction started with denaturation at 94°C for 10 min, followed by 35 cycles at 94°C for 50 sec, 63°C for 50 sec, 72°C for 1 min, then extension at 72°C for 10 min. Amplicons generated were then sequenced.

### PAI-1 polymorphism

Plasminogen activator inhibitor-1 is encoded by the Serpine 1 gene. The 4G/5G polymorphism is located in the promoter region. The 4G/4G genotype leads to a significantly higher PAI-1 plasma concentration than the 4G/5G and 5G/5G genotypes.

Forward primer serpine 1-F2 was 5′-CAC AGA GAG AGT CTG GCC ACG T-3′, reverse primer serpine 1-R4 was 5′-CCA ACA GAG GAC TCT TGG TCT-3′. One hundred fifty ng of genomic DNA was subjected to PCR with 200 μM of dNTP, 1 μM of primers, 1.5 mM of MgCl2, 1.25 U of Taq DNA polymerase (ABI), 1X buffer, in a final volume of 30 μl. The PCR reaction is initiated by denaturation at 94°C for 10 min, followed by 30 cycles at 94°C for 1 min, 63°C for 1 min, 72°C for 2 min, and extension at 72°C for 10 min. The PCR amplicons were digested by restriction enzyme Bs1I for 3 hours followed by separation in a 3% agarose gel [24].

## Results

One hundred sixty-one patients between the ages of 14 and 86 presented with AE (Table 1). Sixty-four patients were male and 97 were female with a mean age of 51 years. Eighty-one percent of patients were of European descent, 15 percent were South Asian, and 4 percent were African Caribbean. The cause of AE was identified in 59 (37%) cases: 3 (2%) patients had a low plasma C1-INH and C4 levels; 20 (12%) were ACE inhibitor-induced;12 (7%) were associated with autoimmune disorders; 7 (4%) were associated with malignancy; and 17 (11%) were related to use of NSAIDs. In the remaining 102 (63%) patients, the cause of AE was idiopathic. Atopy was not identified as a trigger of angioedema in any of the patients. From the entire cohort 67 (42%) patients experienced associated urticaria with their AE which may be typical chronic spontaneous urticaria, while the remaining 94 (58%) did not. In all patients the urticaria was a remote event and not considered related to the angioedema with which they presented.

**Table 1:** **Demographics of patients with angioedema**

		**Entire cohort**	**Subgroup of patients with genetic testing**
Number of patients		**161**	**52**
Mean age (years)		**50.6**	**52.3**
Gender	Male	**64 (39.8%)**	**22 (42.3%)**
	Female	**97 (60.2%)**	**30 (57.7%)**
Presumed etiology	↓ C1-INH, C4	**3 (1.9%)**	**0 (0%)**
	ACE-inhibitor	**20 (12.4%)**	**3 (5.8%)**
	Malignancy	**7 (4.3%)**	**3 (5.8%)**
	Autoimmune	**12 (7.5%)**	**5 (9.6%)**
	NSAID	**17 (10.6%)**	**2 (3.8%)**
	Idiopathic	**102 (63.4%)**	**39 (75%)**

C1-INH, C1 inhibitor; ACE-inhibitor, Angiotensin converting enzyme-inhibitor; NSAID, Nonsteroidal anti-inflammatory drug.

Fifty-two patients out of 161 had genotyping performed. In this group, there were 22 males and 30 females ranging from 24 to 88 years old, with a mean age of 53 years. As seen in Table 2, 13 of the 52 (25%) patients had an identifiable cause: 3 were associated ACE inhibitor use, 3 with malignancy, 5 with autoimmune disease, 2 with NSAID use, and no patients with low C1-INH and C4 levels. In these patients with a known etiology there were a total of 7 mutations/polymorphisms in 6 individuals: 2 in APP (SNP -2399C > A, dbSNP: rs3788853), 1 in ACE (insertion/deletion I/D polymorphism, dbSNP: rs1799752), 2 in PAI-1 (5G/5G polymorphism), and 1 in both ACE and PAI-1.The deletions g.2953-3127 in APP and c.1032C > A or c.1032C > G in factor XII were not detected in any patient. Those with idiopathic AE comprised the remaining 39 (75%) patients. Of these patients, 11 had a genetic variant in APP (SNP -2399C > A, dbSNP: rs3788853), 8 in ACE, 10 in PAI-1, and none in APP (deletion g.2953-3127del) or factor XII. These 29 genetic variants were present in 22 of the idiopathic patients, as 7 of the patients had variants in 2 different genes. Thus 22 of the 39 patients with idiopathic AE had a mutation or polymorphism in one of the studied genes. Of all 52 patients tested, 13 (25%) patients had a specific genetic variant in APP (SNP -2399C > A, dbSNP: rs3788853), 10 (19%) in ACE, 13 (25%) in PAI-1, and none in APP (deletion g.2953-3127del) or Factor XII. Of the entire cohort, genetic variants in multiple genes were present in 9 patients.

**Table 2:** **Patients with a specific genetic variant categorized by presumed etiology**

**Genetic variant**	**APP (deletion)**	**APP (SNP)**		**ACE**	**PAI-1**	**Factor XII**	Total number of patients with at least one genetic variant*
**Presumed etiology (n)**	**(deletion)**	**(C/A)**	**(A)**	**(I/I polymorphism)**	**(5G/5G polymorphism)**	**(C/A, A, C/G, or G)**	
**ACE-inhibitor (3)**	**0**	**0**	**0**	**0**	**1 (1.9%)**	**0**	**1 (1.9%)**
**Malignancy (3)**	**0**	**1 (1.9%)**	**0**	**0**	**0**	**0**	**1 (1.9%)**
**Autoimmune (5)**	**0**	**0**	**0**	**2 (3.8%)**	**2 (3.8%)**	**0**	**3 (5.8%)**
**NSAID (2)**	**0**	**0**	**1 (1.9%)**	**0**	**0**	**0**	**1 (1.9%)**
**Idiopathic (39)**	**0**	**7 (13.5%)**	**4 (7.7%)**	**8 (15.4%)**	**10 (19.2%)**	**0**	**22 (42.3%)**
**Total (52)**	**0**	**8 (15.4%)**	**5 (9.6%)**	**10 (19.2%)**	**13 (25%)**	**0**	**28 (53.8%)**

APP, Aminopeptidase P; ACE, Angiotensin-converting enzyme; PAI-1, Plasminogen-activator inhibitor-1; NSAID, Nonsteroidal anti-inflammatory drug; SNP, Single nucleotide polymorphism. Specific genetic variants were tested in the following genes: APP(deletion g.2953-3127del); APP, (SNP -2399C > A, dbSNP: rs3788853); ACE(insertion/deletion I/D polymorphism, dbSNP: rs1799752)); PAI-1(4G/5G polymorphism); Factor XII( mutations c.1032C > A or c.1032C > G).*Some patients had a genetic variant in multiple genes. Note: only significant genetic variants are reported, as indicated in title row.

*Abbreviations:* *AE* angioedema, *C1-INH*, C1-inhibitor, *ACE* angiotensin converting enzyme, *NSAID* nonsteroidal anti-inflammatory drug, *HAE* hereditary angioedema, *PAI-1* plasminogen-activator inhibitor-1, *APP* aminopeptidase P.

## Discussion

Several classifications of AE have been suggested over the last few decades [10,17,25-27]. These include C1-INH deficiency, use of medications such as ACE inhibitors or NSAIDs, as well as autoimmune conditions and malignancies. Recently, a new classification of AE has been proposed by an international group of experts [10]. It is based on the pattern of presentation (familial or sporadic) and level of C1-INH (normal or low). In some cases, the underlying cause can be identified. Once the offending medication has been discontinued or the underlying disease has been treated, the AE typically resolves, thus confirming the causative factor. However, in many cases no underlying cause is found and the AE is labeled as idiopathic. This of course makes treatment decisions more difficult as there is no specific treatment available. In our cohort, 63% of patients had idiopathic AE; however, while the proportion of patients with idiopathic AE is not clearly reported in the literature a relatively high rate is suspected by many centers [10]. The underlying etiology should help guide treatment decisions. Therefore, discovering the etiology in those with idiopathic AE could lead to better targeted therapeutic options in these patients. We tested 52 patients with AE for genetic variants in genes encoding 4 proteins involved in the regulation of bradykinin generation or catabolism. Fifty-six percent of patients with idiopathic AE had at least one genetic variant. Given these findings we believe that a genetic predisposition could be considered in some patients with AE: the presence of mutations or polymorphisms in factors involved in generation and/or biodisposition of kinins.

Our cohort of 161 patients revealed that most present with AE not related with C1-INH deficiency. Indeed, only three patients demonstrated acquired angioedema with diminished levels of C1-INH. In all three cases the AE was linked to lymphoma and they each had a negative family history for angioedema. The vast majority of patients are thus without effective specific etiology-based treatment for either prophylaxis or acute episodes. The presence of the genetic abnormalities we tested for could help explain the occurrence of AE in many patients and help better establish treatment options that could supplement standard treatment. For example, a specific inhibitor of kallikrein (Ecallantide) and/or a bradykinin B2 receptor antagonist (Icatibant) could be used in patients with AE not caused by C1-INH deficiency. Some reports show that use of C1-INH concentrates or Icatibant in patients with AE not linked to C1-INH deficiency have mixed results. Response to treatment may depend on the underlying mutation/polymorphism [10]. In those with genetic variants of PAI-1, reduced or dysfunctional protein could lead to more active fibrinolysis and presumably increased generation of bradykinin. Antifibrinolytic agents, such as tranexamic acid and ε-aminocaproic acid, could be considered as a treatment of choice in these patients. In the case of HAE or acquired AE, these medications could supplement C1-INH concentrates as the clinical presentation, severity, and frequency of AE attacks vary in patients with C1-INH deficiency, which may be explained by genetic variants present in those patients. Angioedema occurs in association with various clinical settings but the risk and severity of attacks could be linked to the presence of an allelic variant in one of the molecules involved in bradykinin generation and/or catabolism. As new genetic variants are discovered, more therapies can be developed to target the implicated proteins.

It is possible that the genetic variants that we identified may actually be risk factors to developing AE. For instance, the presence of the 5G/5G polymorphism associated with a low level of PAI-1 leading to higher level of generation of plasmin may predispose someone to developing AE in the presence of therapy with ACE inhibitors which decrease the biodisposition of kinins. Similar rationale can be applied to those with AE secondary to malignancy, autoimmune disease, or NSAID use. Polymorphisms in APP and ACE lead to decreased bradykinin biodegradation, whereas gain-of-function mutations in factor XII increase its production.

One limitation of our study is that a control group was not tested. Evaluation of the frequency of gene variant occurrence in a control population would be helpful in assessing a potential role in development of AE. However, allele frequencies are highly influenced by ethnicity, as reported for some variants we tested for such as PAI-1 [20,28] and ACE [29]. Only specific polymorphisms/mutations in APP, ACE, PAI-1, and factor XII were tested. It is likely that other regions of these genes or other genes involved in the kinin-generating pathway may have variants affecting protein function that can lead to altered regulation of bradykinin. Based on European populations, for the SNP –2399C > A (rs3788853), the frequency of allele C is 73%, while the frequency of allele A is 26% [30]. For the 4G/5G PAI-1 gene polymorphism, the frequency of 4G is 50% and the frequency of 5G is 50% [20,28]. For the I/D polymorphism (rs1799752) in the ACE gene, the frequency of D is 50% in Northern Europeans and 52% in Southern Europeans, respectively, while the frequency of I is 50% in Northern Europeans and 48% in Southern Europeans, respectively [29]. In our patients presenting with angioedema these genetic variants, by themselves or in association with other unknown cofactors, such as stress or trauma, can be responsible for their clinical manifestations. This is an investigative study that probes novel genetic mutations and polymorphisms and requires further work with a larger population size. Nonetheless, this research directs future studies on this poorly understood group of disorders of bradykinin metabolism. These results demonstrate genetic variants that would affect patients presenting with angioedema.

## Conclusions

Our study explored the possibility that defects in genes encoding proteins involved in kinin generation or catabolism other than C1 inhibitor may be involved in angioedema. This data suggests that a high proportion of patients with angioedema may have various defective protein functions leading to sustained bradykinin effects upon triggering of its generation in AE. Functional studies addressing this possibility would be warranted.

Our preliminary observations support the hypothesis that the occurrence of AE attacks could be facilitated by a variety of a variety of variants in genes encoding proteins involved in generation and/or biodisposition of kinins known to be mediators of this clinical syndrome. A high proportion of patients may present with genetic variants, which needs to be verified in further studies.

In the future, patients with unusual phenotypes of AE, including those with reduced C1-INH function, could be investigated for gene variants associated with bradykinin generation and degradation that are likely to contribute to their clinical syndrome. Results of such studies could help understand the pathophysiology of this clinically variable syndrome. This could eventually lead to more effective personalized therapy and prevention of attacks.

## Abbreviations

AE: Angioedema
C1-INH: C1 inhibitor
ACE: Angiotensin converting enzyme
NSAID: Nonsteroidal anti-inflammatory drug
HAE: Hereditary angioedema
PAI-1: Plasminogen-activator inhibitor-1
APP: Aminopeptidase P
dbSNP: Single nucleotide polymorphism database
SNP: Single nucleotide polymorphism
